# A hybrid electrochemical device based on a synergetic inner combination of Li ion battery and Li ion capacitor for energy storage

**DOI:** 10.1038/srep41910

**Published:** 2017-02-07

**Authors:** Jun-Sheng Zheng, Lei Zhang, Annadanesh Shellikeri, Wanjun Cao, Qiang Wu, Jim P. Zheng

**Affiliations:** 1 Clean Energy Automotive Engineering Center, Tongji University (Jiading Campus), 4800 Caoan Road, Shanghai 201804, China; 2 School of automotive studies, Tongji University (Jiading Campus), 4800 Caoan Road, Shanghai 201804, China; 3 Department of Electrical and Computer Engineering, Florida A&M University-Florida State University College of Engineering, Florida State University, Tallahassee, FL 32310, USA

## Abstract

Li ion battery (LIB) and electrochemical capacitor (EC) are considered as the most widely used energy storage systems (ESSs) because they can produce a high energy density or a high power density, but it is a huge challenge to achieve both the demands of a high energy density as well as a high power density on their own. A new hybrid Li ion capacitor (HyLIC), which combines the advantages of LIB and Li ion capacitor (LIC), is proposed. This device can successfully realize a potential match between LIB and LIC and can avoid the excessive depletion of electrolyte during the charge process. The galvanostatic charge-discharge cycling tests reveal that at low current, the HyLIC exhibits a high energy density, while at high current, it demonstrates a high power density. Ragone plot confirms that this device can make a synergetic balance between energy and power and achieve a highest energy density in the power density range of 80 to 300 W kg^−1^. The cycle life test proves that HyLIC exhibits a good cycle life and an excellent coulombic efficiency. The present study shows that HyLIC, which is capable of achieving a high energy density, a long cycle life and an excellent power density, has the potential to achieve the winning combination of a high energy and power density.

The automotive industry has made remarkable contributions to the world economy and human mobility^1^, but excessive consumption of fossil fuels as an energy source for automobiles leads to serve environmental pollution and greenhouse gas emissions^2^. It is well known that the electric vehicles (EVs) is one of the most promising alternatives with a lot of advantages over the conventional gas powered automobiles^3^. Therefore, to develop available energy storage systems (ESSs) are extremely critical in ensuring the success of the EVs^4^.

Li ion battery (LIB) and electrochemical capacitor (EC) are considered as the most widely used ESSs for EVs because they can deliver a high energy density and a high power density, respectively. But they stand at two ends of the spectrum and presently it is impossible for a LIB or EC to achieve the twin demands of high energy density as well as high power density on their own^5^, owing to the intrinsic chemistry restrictions and the kinetics involved. In this case, an emerging concept of hybrid systems has been extensively investigated and been reported for a long period with different approaches, such as Ni-MH battery and EC hybrid system, LIB and EC hybrid system. These external hybrid systems (eHS) with complex structure achieve a quite limited improvement of energy density in comparison with traditional EC. Hence, more work transfer into developing higher performance hybrid system by an internal hybridized method denoted by inner hybrid systems(iHS).

In our previous work, we have reported a iHS novel of lithium ion capacitor (LIC) by replacing the conventional AC negative electrode with a hard carbon (HC) negative electrode covered by a stabilized lithium metal powder (SLMP) layer^6^,^7^,^8^. The added SLMP can increase the open circuit voltage (OCV) and ensures a low electrolyte consumption when it is charged. This LIC is capable of storing approximately 4 times more energy than conventional ECs and simultaneously maintain the benefits of a high power density. However, this device still can’t satisfy the market requirement completely.

Based on the analysis of the chemical properties of LIB and LIC, in the present work, we proposed a new hybrid electrochemical device, which synergistically combines the advantages of LIBs and LICs and has the potential to significantly improve the performance of ESSs. This device, namely, hybrid Li ion capacitor (HyLIC) consists of a positive electrode with a two-side structure: a side of lithium ion intercalating metal oxide particles combined with a side of AC; and a negative electrode consisting of HC loaded with a layer of SLMP, the schematic being shown in Fig. 1. With this special structure, the negative electrode with same structure as LIC negative electrode can keep a more negative potential, limit the electrolyte consumption and provide a higher energy density. The positive electrode is an inner combination of LiCoO_2_ (LIB material) and AC (LIC material). The advantages of this configuration are that at low operating rates, the device will reflect the characteristic of LIB, providing a high energy density. While at high operating rates, it will exhibit the features of LIC, providing a high power density. We present here our study of the proposed energy storage device, HyLIC, which has shown a high potential to satisfy the twin demands of high power and energy density facing to different operation condition, favorably demonstrating a diagonal characteristic on the Ragone plot.

## Experimental Section

Commercial active materials were used for both the positive and negative electrodes as received. The slurry mixture of the negative electrode was made of hard carbon (HC, Carbotron P(J), Kureha, Japan) and 5% polyvinylidene fluoride (PVDF) in N-methyl-2-pyrrolidone (NMP) as a binder by the mass ratio of 92:8. After the slurry was prepared, the doctor blade method was employed to spread the prepared slurry onto a 10μm thick Cu foil substrate.

The positive electrode based on AC was prepared by coating a slurry mixture of activated carbon (AC, AB-520, MTI Corporation) and PVDF binder in NMP solvent by the mass ratio of 9:1. The positive electrode based on LiCoO_2_ (EQ-Lib-LCO, MTI Corporation) was prepared by mixing 85% active material, 10% carbon black, and 5% PVDF in NMP. After the slurry was prepared, it was coated onto an Al foil substrate by the mentioned doctor blade method. Finally, all electrodes were dried at 120 °C for 12 h to remove the solvent (NMP) and water. After the electrodes were dried, a hot-roll pressing process was employed to prepare electrodes sheets of desired thickness. All the electrode sheets were kept in the dry room and punched out into circular electrodes with a diameter of 1.27 cm. The average mass loading of HC, AC and LiCoO_2_ on electrode was 13.22 mg · cm^−2^, 4.2 mg · cm^−2^ and 10.9 mg · cm^−2^, respectively.

The stabilized lithium metal powder (SLMP), used as obtained from FMC Lithium (avg. powder size ~10–200 nm), was applied onto the surface of the prefabricated HC negative electrodes in an argon atmosphere in a glove box. Based on the designed capacity of the prefabricated HC electrodes, appropriate quantity of SLMP was loaded, spread evenly and pressed onto the anode electrode, forming a uniform thin layer of SLMP.

The powder X-ray diffraction (XRD, Rint-1100, Rigaku, Japan) measurement using Cu Kα radiation was employed for XRD study of different electrodes. Nuclear Magnetic Resonance (NMR) spectroscopy was performed to analyze the SLMP loading effect on hard carbon, with the spectra recorded using the prepared samples in a horizontal position with respect to the external applied magnetic field. ^7^Li NMR spectra were acquired at 233 MHz using a Bruker Avance I 600 MHz (14.1 Tesla) spectrometer.

Two-electrode Swagelok-type cells were assembled to characterize the performances of cells. Cells were charged and discharged under a constant current density of 0.4 mA · cm^−2^ in the potential range ~2.0 to 4.1 V. The electrolyte was 1 M LiPF_6_ in ethylene carbonate (EC): dimethyl carbonate (DMC) at a ratio of 1:1 by weight (LP30, SelectiLyte^TM^, Merck Electrolyte). The electrochemical impedance spectrum (EIS) for different cells were also studied in the frequency ranging from 1 MHz to 0.1 Hz by a Gamry Instruments. The recorded EIS spectra were fitted using Gamry Echem Analyst program. For the HyLIC cycle life study, the cell was charged and discharged under constant current density of 4 mA∙cm^−2^ in the potential range ~2.0 to 4.1 V.

The different electrochemical devices assembled with different positive electrodes of LiCoO_2_, LiCoO_2_/AC and AC are referred to as LIB, HyLIC and LIC, respectively. The energy density and power density are calculated based on electrode material weight (active materials and binder) of both the negative and the positive electrodes.

## Results and Discussion

**Figure 2:**
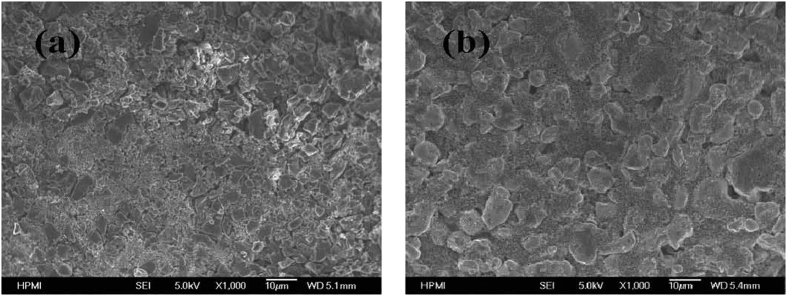
Typical SEM images of cathode of Li ion device. (**a**) AC cathode, (**b**) LiCoO_2_ cathode.

The SEM images of the positive AC and LiCoO_2_ electrodes are presented in Fig. 2. It can be observed that the AC electrode shows an irregular shape with sharp edges of the particle sizes ~1–3 μm. And the LiCoO_2_ particles with a particle size ~1 um were covered by carbon black.

**Figure 3:**
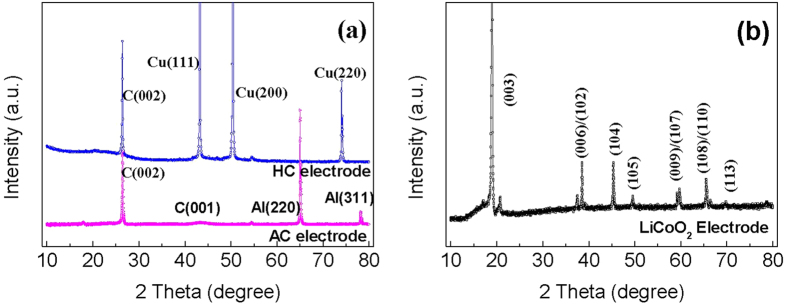
XRD profiles of different electrodes: (**a**) AC eltrode and HC electrode, and (**b**) LiCoO2 electtode.

Figure 3 exhibits the XRD pattern of different electrodes. As mentioned above, the negative and positive electrodes are supported on copper and aluminum, respectively. Strong Al and Cu characteristic peaks can be found from Fig. 3. A strong (002) diffraction peak at 2θ ~ 26.5° (Fig. 3a) can be attributed to the graphite-like structure [C(002)] of HC and AC and the weak C(100) peak corresponding to the formation of a disordered carbon material^9^,^10^. On the other hand, the major characteristic peaks (Fig. 3b) of rhombohedra LiCoO_2_ (2θ = 19.0°, 38.6°, 39.2°, 45.4°, 49.6°, 59.2°, 59.9°, 65.5°, 66.5° and 69.8°) that are associated with the (003), (006), (012), (104), (105), (009), (107), (108), (110) and (113) reflections are observed in the 2θ range from 10° to 75°, respectively^11^. The clear split of doublets at (006)/(012) and (018)/(110) reflections for all samples demonstrates that these materials have well-defined layered structure.

**Figure 4:**
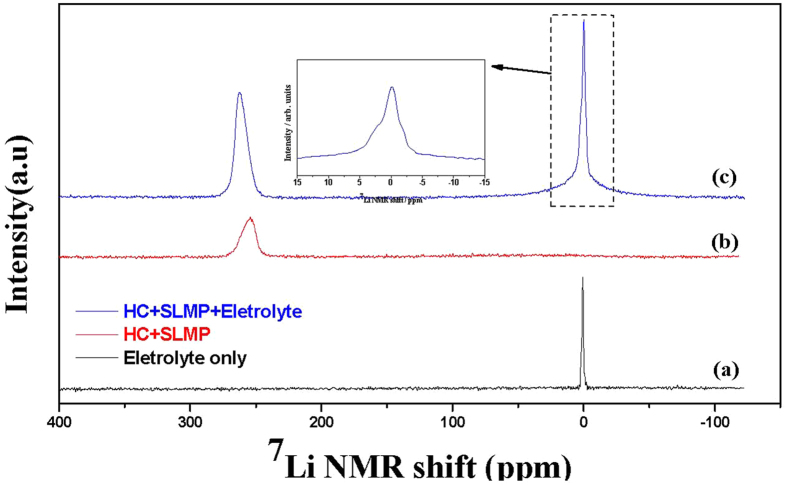
^7^Li NMR spectra of samples containing (**a**) the electrolyte (1 M LiPF6 in EC: DMC at a ratio of 1:1) only, (**b**) SLMP loaded HC electrode only and (**c**) SLMP loaded HC electrode with the electrolyte. Inset: expansion of the spectrum in (**c**) from +15 to −15 ppm.

We further investigated the SLMP loading on HC anode electrode using NMR spectroscopy. Three samples were prepared, sample-(a) with the electrolyte (1 M LiPF_6_ in EC: DMC at a ratio of 1:1) only, sample-(b) with the SLMP loaded HC electrode only and sample-(c) with the SLMP loaded HC electrode and the electrolyte. The recorded spectra are shown in Fig. 4.

The ^7^Li signal from the electrolyte sample-(a) appears as a sharp narrow peak around 0 ppm due to the rapid and isotropic tumbling of the ^7^Li nucleus in the liquid electrolyte phase, which averages out all the broadening inducing magnetic interactions (quadrupolar and dipolar). The sample-(b) shows only the metallic-peak around 270 ppm, due to the SLMP layer on anode, consistent with the assignment in other works^12^,^13^,^14^ and no peak is observed for the electrolyte due to absence of it in the sample. This lithium metal contribution is relative to the electrolyte species and the shift is known as Knight Shift, which is a characteristic of the ^7^Li nuclei. Furthermore, the NMR spectrum from sample-(c) has a broadened electrolyte peak at the base around 0 ppm and the metallic-peak around 270 ppm. The broadening at the base is due to the presence of li-metal in addition to the carbon surface, both in contact with electrolyte. An additional peak around 2.5 ppm is also observed, as seen in the inset of Fig. 4, which is further attributed to the initial li-intercalation in the HC electrode, consistent with earlier assignment^14^. This li-intercalation is due to the shorting effect of the lithium metal from SLMP with the HC surface on injection of electrolyte.

**Figure 5:**
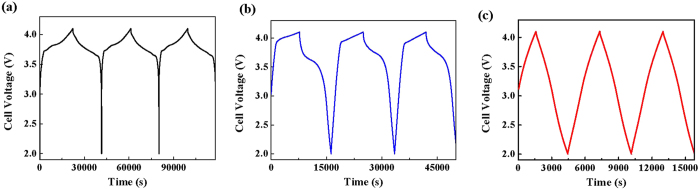
Voltage profiles of cells with a charge and discharge current of 0.4 mA · cm^−2^: (**a**) LIB, (**b**) HyLIC, and (**c**) LIC.

Figure 5 shows the voltage profiles for LIB, LIC and HyLIC, which were galvanostatically charged and discharged in the voltage range of 4.1–2.0 V under a constant current of 0.4 mA · cm^−2^. OCVs of all cells in pristine state are higher than 2.0 V. In contrast, for the traditional EC or hybrid capacitor, pristine cell’s OCV is ~0.0 V. The higher OCVs displayed by the assembled LIC and HyLIC devices is due to the presence of SLMP, which was applied on the anode, as mentioned earlier. It acts as a lithium reservoir and its presence has the beneficial effect of reducing the over-depletion of electrolyte during solid-electrolyte-interface (SEI) formation, and hence, can improve the energy density of LIC and HyLIC and cycling stability.

Also, it is important to note that LIB, LIC and HyLIC have the same operating potential range of 2.0–4.1 V. As mentioned before, one of the most important properties of HyLIC is the potential matching of LIB and LIC devices, while combining their advantages together in a single device. As expected, LIB is characterized by its typical voltage profile with insertion plateau in the range 3.6–4.0 V, consistent with the battery cathode material LiCoO_2_, Fig. 5(a). Further, the LIC, with AC cathode, is distinctly characterized by its linear voltage profile, Fig. 5(c). The HyLIC, which consists of the bi-material (LiCoO_2_ + AC) segment electrode as cathode, shows features typically observed for the two materials. The typical linear voltage profile characteristic of the capacitive component (AC) is clearly observed at the beginning of discharge, confirming that the main part of the current went through AC during this time. And a voltage plateau can be located in the later part of the discharge profile, which means that during this time period, the majority of the current was passing through LiCoO_2_. The gravimetric energy densities of LIB, HyLIC and LIC were observed to be 344.1 Wh · kg^−1^, 149.2 Wh · kg^−1^, and 58.7 Wh · kg^−1^, respectively. Hence, at a relatively lower rate of 0.4 mA · cm^−2^, LIB demonstrated an energy density 5 times higher than that of LIC, while that for HyLIC is ~3 times higher.

**Figure 6:**
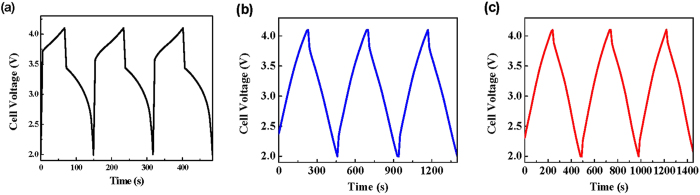
Voltage profiles of cells with various positive electrodes with a current of 4 mA · cm^−2^: (**a**) LIB, (**b**) HyLIC, and (**c**) LIC.

To further understand the performance of HyLIC under high power demands, the cells were galvanostatically charged-discharged at a much higher rate of 4 mA · cm^−2^ (10 times that shown in Fig. 5). Figure 6 shows the voltage profiles of the first three cycles for LIB, HyLIC and LIC in the voltage range of 4.1–2.0 V under a constant current density of 4 mA · cm^−2^. At this higher rate, the energy densities of LIB, HyLIC and LIC were observed to be 6.0 Wh · kg^−1^, 31.1 Wh · kg^−1^, and 46.2 Wh · kg^−1^, respectively. Clearly, HyLIC outperforms the LIB at high rates. These results clearly demonstrate that HyLIC combines the high energy density characteristic of LIBs at lower rates with that of LICs at high rates, hence presenting a synergetic combination of these two devices.

As we can see from the cell voltage profiles presented in Figs 5 and 6, the linear part (2.0 V–3.6 V) represents the contribution from the capacitive material (AC) which plays the dominant role, while the plateau part (3.6 V–4.0 V) shows that the major contribution came from the battery electrode material (LiCoO_2_). Significantly, when the current is comparatively low at 0.4 mA · cm^−2^ in Fig. 5, the plateau is a major feature of the voltage profile of HyLIC, indicating that LiCoO_2_ contributed in bulk compared to AC. However, when the current reaches to 4 mA · cm^−2^, ~90% capacity in the voltage profile of HyLIC comes from the capacitive material (AC), as evidently observed from the linear characteristic of the voltage profile. These observations reveal that at low current density, the battery materials play the dominant role while at comparatively high current density, the capacitive materials are of much more importance.

**Figure 7:**
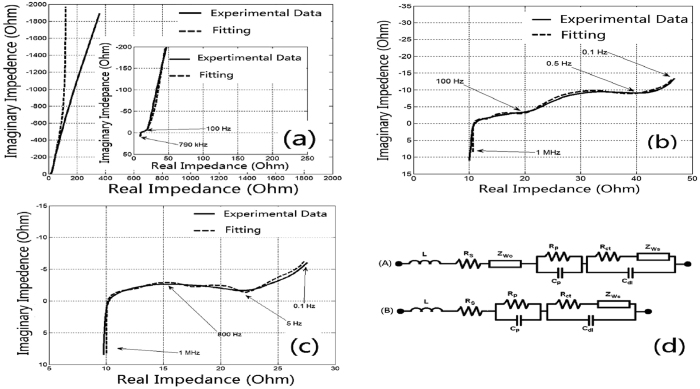
Nyquist plot and fitted EIS data of (**a**) LIC, (**b**) LIB, and (**c**) HyLIC, and (**d**) Equivalent circuit models of (A) LIC, (B) LIB and HyLIC cells.

The electrochemical impedance spectra (EIS) were recorded for LIC, LIB and HyLIC after preliminary equilibrium and fitted using the electric equivalent circuit model^11^, and are presented in Fig. 7. Table 1 lists the corresponding fitted resistance parameters. The spectra were recorded in the range from 1 MHz to 0.1 Hz with a signal amplitude of 10 mV. All the EIS spectra recorded exhibited depressed semicircles in various degrees in the high to middle frequency regions and a linear low frequency region. The electrolyte resistance and contact resistance of the electrode are represented by R_S_. The high frequency semicircle relates to the lithium ion migration resistance in the passivation layer. R_P_ and C_P_ are the passivation layer parameters defined by the SLMP materials left on the surface of the HC during initial cycling. This passivation layer is likely the leftover Li material that originally formed the shell of the SLMP and the initial SEI formed on HC. The middle frequency region semicircle relates to the charge transfer resistance (R_CT_) and the low frequency region straight line represents the Li ion diffusion impedance (Warburg element).

Table 1 reveals that charge transfer kinetics (R_ct_/C_dl_) are similar in value. This may be due to the charge transfer of Li ions in the HC intercalating in and out of the Li_1−x_C_6_ matrix in all the three cell types. The LIC should behave like a Warburg open-circuit element (R_Wo_ and T_Wo_) while the LIB and HyLiC should behave like a short-circuit Warburg element (R_Ws_ and T_Ws_). From literature^23^, LIB generally has a lower diffusion coefficient for Li ions than in LIC, leading to the conclusion that the short-circuit Warburg element seen in Fig. 7b for LIB corresponds directly to the LiCoO_2_ cathode material. This is why a high impedance value for the R_Ws_ parameter is observed for the LiB. For the HyLiC, we obviously can find two electrochemical processes in the form of two well defined depressed semicircles in the high to mid frequency regions followed by a low frequency linear region, which confirms the hybrid nature of operation of LIB and LIC in HyLIC, with an expected intermediate Warburg short-circuit parameter values (R_Ws_ and T_Ws_) as seen in Table 1.

**Figure 8:**
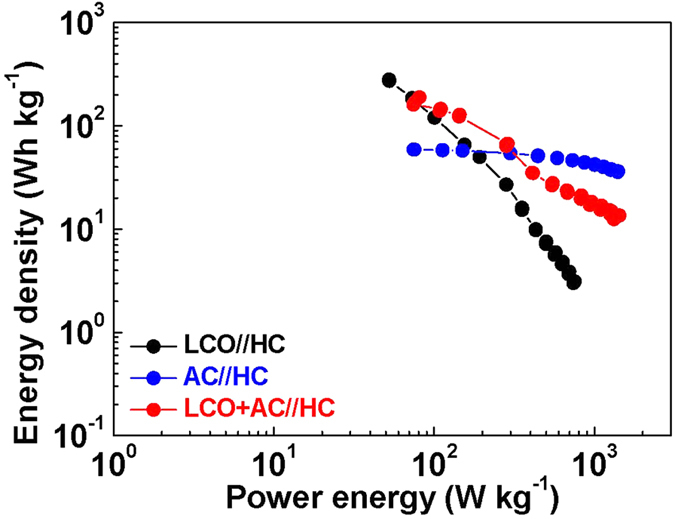
Ragone plots of LIB, LIC and HyLIC based on different cathodes on the gravimetric bases.

Furthermore, the energy density and the power density of the HyLIC were investigated and compared to that of LIB and LIC devices. The different devices (LIB, LIC and HyLIC) were discharged under constant power mode and Fig. 8 shows the resulting Ragone plot based on the weight of activate materials. Predictably, the gravimetric energy density decreases with the increase in power density for all three cells, which means it is a greater challenge to realize an energy storage device which can satisfy the dual demands of a high energy density as well as a high power density. On the other hand, LIC has a much lower decrease rates in comparison with LIB as the power density increases indicating LIC has better high-rates performance. This HyLIC combining both advantages of LIB and LIC with a middle decrease rate, achieve highest energy density when the power energy is from 80 to 300 W kg^−1^. This critical character means HyLIC is a best choice to apply in some fields, such as the power source of start/stop system for hybrid vehicle. When the power densities are less than 80 W · kg^−1^, LIB demonstrates a highest energy density than others. While with a power density higher than 300 kW · kg^−1^, LIC performs better than others with the highest energy density.

**Figure 9:**
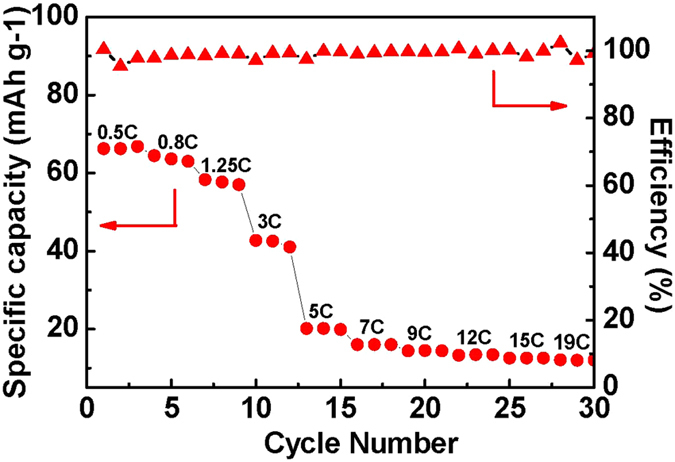
Multi-rates performance of HyLIC.

As we know, electrical energy in LIBs is generated by conversion of chemical energy via oxidation/reduction reactions. While in LICs, energy is delivered via orientation of electrolyte ions at the electrode/electrolyte interface. For HyLIC, the use of the combination electrode having two kinds of active materials (AC + LiCoO_2_) has the advantage of both the capacitor and battery materials by bringing together the two different energy-storing mechanisms, namely double layer formation and redox/faradic reactions. Figure 9 displays the multi-rates performance of HyLICs, and this HyLIC delivers a high specific energy density of 66.7 mAh g^−1^ at relative low rate of 0.5 C while still remaining 12.5 mAh g^−1^ at 19C. Correspondingly, in a situation that demands low power/rates, the HyLIC can perform the function of LIB by responding with a high energy density, outperforming the LIC, as clearly seen in Fig. 8. With the increase of specific power density, the specific energy density of three cells tends to decrease at different rates. Eventually, LIB exhibits the lowest specific energy density of 3 Wh · kg^−1^ at high specific power density of 1000 W · kg^−1^. The properties of LIC are quite different from that of LIB. The specific energy density of LIC is relatively stable with the increase of specific power density and at ~1000 W · kg^−1^, the specific energy density decreases to 42 Wh · kg^−1^. Most significantly, the HyLIC cell combines the former two features. At first, the specific energy density of HyLIC stays comparatively high as predicted earlier, and subsequently its rate of decrease with the increase of specific power density remains intermediate to LIC and LIB. For the HyLIC, an energy density of 21 Wh · kg^−1^ could be achieved at a delivered power density of 1000 W · Kg^−1^. Table 2 lists the performances of LIB, LIC and HyLIC different energy and power demands, confirming the diagonal nature of HyLIC’s performance on the Ragone Plot.

Clearly, from Table 2, HyLIC can respond with a higher energy density than LIC during low power demands, and exhibits a higher power density than that of LIB during low energy demands. These results confirm that the proposed new HyLIC has a better balance and synergy on power and energy and a clear advantage over batteries and electrochemical capacitors.

**Figure 10:**
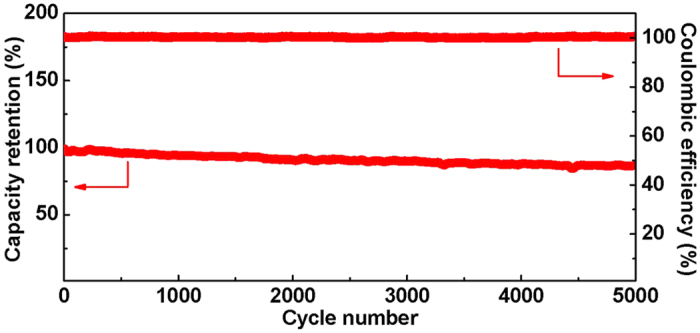
The specific energy density and the energy efficiency as a function of cycle number for HyLIC.

Cycle life study was performed for HyLIC, and the Fig. 10 shows the specific energy density retention and round-trip energy efficiency as a function of cycle number for HyLIC at 30C. It can be observed from Fig. 10 that in the 5000 cycles, there is negligible degradation in the discharge energy density. The HyLIC still demonstrates a capacity retention of >87% of the maximum discharge specific energy density after 5000 cycles. The coulombic efficiency was observed to be near to 100% during the entire cycles. These results show that HyLIC has a stable capacity retention and excellent coulombic efficiency.

## Conclusion

To combine the advantages of LIB and LIC, a novel electrochemical energy storage device HyLIC was developed. The voltage profiles for cells show that at low current rate (power demand of 60 W · Kg^−1^), the new device, HyLIC exhibited a higher energy density of ~150 Wh · Kg^−1^ against only ~60 Wh · Kg^−1^ for LIC. At a high current rate (power demand of 1000 W · Kg^−1^), this device delivers a higher energy density of ~21 Wh · Kg^−1^ against only ~3 Wh · Kg^−1^ for LIB. The Ragone plot proves that this novel device has the advantages of LIB and LIC and achieve a highest energy density in the power density range of 80 to 300 W kg^−1^. HyLIC can make a balance between energy and power; and therefore, can satisfy the demand of a high energy density and a high power density for ESSs.

## Additional Information

**How to cite this article:** Zheng, J.-S. *et al*. A hybrid electrochemical device based on a synergetic inner combination of Li ion battery and Li ion capacitor for energy storage. *Sci. Rep.* **7**, 41910; doi: 10.1038/srep41910 (2017).

**Publisher's note:** Springer Nature remains neutral with regard to jurisdictional claims in published maps and institutional affiliations.

## Figures and Tables

**Figure 1:**
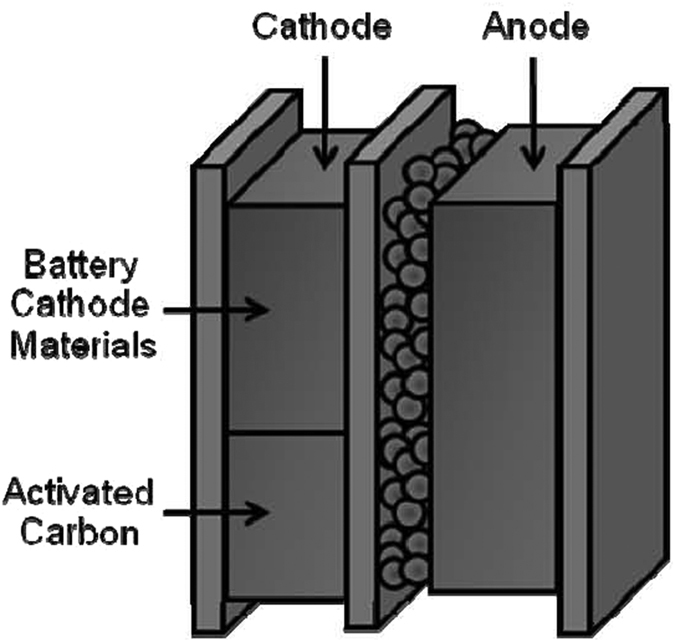
Schematic for a hybrid Li ion capacitor (HyLIC).

**Table 1:** Equivalent Circuit Parameters for LIB, LIC and HyLIC.

Cell	R_S_(Ω)	R_P_(Ω)	C_P_(Ω)	R_ct_(Ω)	C_dl_(F)	R_Ws_(Ω)	T_WS_(ms)	R_Wo_(Ω)	T_Wo_(s)
LIB	10.22	1.62	2.06E-06	3.4	2.61E-05	112.7	927.64	/	/
LIC	10.05	4.16	4.80E-05	2.52	5.95E-06	4.53	8.41	20	6. 87
HyLIC	10.73	2.55	5.51E-06	4.91	7.72E-05	15.87	164.56	/	/

**Table 2:** Power and energy performances of LIB, LIC and HyLIC cells.

Cell	Power Performance W · Kg^−1^	Energy Performance Wh · Kg^−1^
	(at 5 Wh · Kg^−1^)	(at 60 W · Kg^−1^)
LIB	800	340
LIC	3500	60
HyLIC	2000	150
